# Wie vergleichbar sind Daten zur körperlichen Aktivität? Methodische Unterschiede und ihre Bedeutung für evidenzbasierte politische Entscheidungen

**DOI:** 10.1007/s00103-026-04316-5

**Published:** 2026-10-16

**Authors:** David Kossmann, Anna Hirschbeck, Naiiri Khorikian-Ghazari, Moritz Bruno Petzold, Alkomiet Hasan, Astrid Röh

**Affiliations:** 1 Klinik für Psychiatrie, Psychotherapie und Psychosomatik, Medizinische Fakultät, Universität Augsburg, Augsburg, Deutschland Geschwister-Schönert-Str. 1, 86156; 2 Deutsches Zentrum für Psychische Gesundheit (DZPG), Standort München/Augsburg, Augsburg, Deutschland; 3 Department Psychologie, MSB Medical School Berlin, Berlin, Deutschland

**Keywords:** Prävalenzschätzungen, Erhebungsmethoden, Inaktivität, Public Health, Messinstrumente, Prevalence estimates, Assessment methods, Inactivity, Public health, Measurement instruments

## Abstract

Körperliche Aktivität ist eine zentrale Determinante von Gesundheit und spielt eine wesentliche Rolle in der Prävention diverser chronischer und psychischer Erkrankungen. Zahlreiche Studien belegen die positiven Effekte regelmäßiger körperlicher Aktivität auf Morbidität, Mortalität und Lebensqualität. Aktuelle globale Analysen, wie die im *Lancet* publizierte Studie von Strain et al. (2024), weisen für Deutschland im internationalen Vergleich eine hohe Erfüllung der WHO-Bewegungsempfehlungen aus und schätzen den Anteil unzureichend körperlich aktiver Erwachsener auf rund 12 %. Nationale Erhebungen berichten hingegen deutlich höhere Anteile von bis zu 54 %.

Der Diskussionsbeitrag nimmt diese Diskrepanz zum Ausgangspunkt, um Ergebnisse verschiedener Studien zur körperlichen Aktivität gegenüberzustellen und methodisch einzuordnen. Unterschiede in Messinstrumenten, Befragungsmodi, berücksichtigten Lebensbereichen, Operationalisierungen und Modellierungsverfahren können die Prävalenzschätzungen erheblich beeinflussen und die Vergleichbarkeit zwischen Studien einschränken. Selbstberichtete Daten können körperliche Aktivität überschätzen, während objektive Verfahren wie die Akzelerometrie mit eigenen methodischen Herausforderungen verbunden sind.

International modellierte Schätzungen liefern wichtige Daten für länderübergreifende Vergleiche, sollten jedoch bei der Bewertung der nationalen Situation unter Berücksichtigung ihrer methodischen Grundlagen interpretiert werden. Für die Erfassung körperlicher Aktivität besteht weiterhin methodischer Entwicklungsbedarf. Eine Kombination aus Selbstbericht und objektiver Messung könnte perspektivisch dazu beitragen, die Datengrundlage für evidenzbasierte politische Entscheidungen zur Förderung körperlicher Aktivität zu verbessern.

## Abstract (en)

Physical activity is a key determinant of health and plays a crucial role in the prevention of various chronic diseases and mental disorders. Numerous studies demonstrate the positive effects of regular physical activity on morbidity, mortality, and quality of life. Recent global analyses, such as the *Lancet* study by Strain et al. (2024), indicate comparatively high compliance with WHO physical activity recommendations in Germany and estimate the proportion of insufficiently active adults at around 12%. In contrast, national surveys report substantially higher rates of up to 54%.

This discussion article takes this discrepancy as a starting point to compare and methodologically contextualize study findings. Differences in measurement instruments, survey modes, domains considered, operational definitions, and modelling approaches can substantially influence prevalence estimates and limit comparability across studies. Self-reported data may overestimate physical activity, while objective measures such as accelerometry present their own methodological challenges.

Internationally modeled estimates provide valuable data for cross-country comparisons but should be interpreted in light of their methodological foundations when assessing the national situation. Further methodological development is needed in assessing physical activity. Combining self-report and objective measurement could help provide a more robust basis for evidence-based policy decisions to promote physical activity.

## Hintergrund

Körperliche Aktivität gilt als zentraler Faktor für körperliche und mentale Gesundheit und als essenzieller Bestandteil in der Primär- und Sekundärprävention sowie Behandlung zahlreicher somatischer und psychischer Erkrankungen. Eine breite Evidenzbasis nationaler und internationaler Studien belegt, dass regelmäßige körperliche Aktivität signifikante gesundheitliche Effekte erzielt, darunter eine Stabilisierung des Herz-Kreislauf-Systems [1], eine Stärkung des Muskel-Skelett-Systems [2] und eine Reduktion des Risikos für chronische Erkrankungen wie Adipositas [3], Rückenschmerzen [4], Herz-Kreislauf-Erkrankungen [5] sowie bestimmte Krebserkrankungen [6]. Auch in der Prävention und unterstützenden Behandlung von Erkrankungen wie Typ-2-Diabetes [7], Herz- und Kreislauf-Erkrankungen [8] und psychischen Erkrankungen (u. a. Depression, Angsterkrankungen, Suchterkrankungen, Schizophrenie; [9–12]) ist der gesundheitsfördernde Effekt gut dokumentiert. Ein hohes Level an körperlicher Aktivität korreliert zudem konsistent mit erhöhter Lebensqualität [13] und Lebenserwartung [14].

Die Förderung körperlicher Aktivität ist vor diesem Hintergrund eine gesamtgesellschaftliche und gesundheitspolitische Aufgabe von hoher Priorität. Unzureichende körperliche Aktivität ist mit erheblichen individuellen Krankheitslasten sowie volkswirtschaftlichen Belastungen assoziiert, sowohl durch direkte Kosten im Gesundheitswesen als auch durch indirekte Effekte wie Produktionsausfälle und verminderte Erwerbsfähigkeit. Aus Public-Health-Perspektive bedarf es daher einer konsequenten, systemisch angelegten Strategie zur Bewegungsförderung. Ein historisch bedeutsames Beispiel ist die vom Deutschen Sportbund initiierte „Trimm-Dich“-Kampagne (1970–1994), eine der bis dato umfassendsten Gesundheitskampagnen in Deutschland. Durch Trimmpfade, Lauf-Treffs und breite Öffentlichkeitsarbeit wurden Millionen Menschen zu mehr Bewegung motiviert, was das gesellschaftliche Klima zugunsten körperlicher Aktivität veränderte und den Breitensport nachhaltig stärkte [15]. Effektive Bewegungsförderung erfordert ein evidenzbasiertes Vorgehen. Politische Entscheidungsträger sind auf valide epidemiologische Daten angewiesen, um Bedarfe zu identifizieren, Fortschritte zu messen und Prioritäten zu setzen. Die Entwicklung internationaler Standards und Surveillance-Systeme bildet daher ein zentrales Fundament für wirksame Präventionsstrategien.

Vor diesem Hintergrund veröffentlichte die Weltgesundheitsorganisation (WHO) im Jahr 2004 erstmals eine globale gesundheitspolitische Strategie zu Ernährung, körperlicher Aktivität und der Prävention nichtübertragbarer Erkrankungen. Damit sollten politische und gesellschaftliche Rahmenbedingungen für gesunde Ernährung und mehr körperliche Aktivität geschaffen werden. Darauf aufbauend veröffentlichte die WHO 2010 konkrete altersgruppenspezifische Empfehlungen zur körperlichen Aktivität und schuf damit einen internationalen Orientierungsrahmen für Wissenschaft, Praxis und Politik. Diese wurden zuletzt 2020 auf Basis aktueller Evidenz überarbeitet und betonen inzwischen auch die gesundheitsförderlichen Effekte kurzer Bewegungsintervalle [16]. Die derzeit gültigen Empfehlungen raten gesunden Erwachsenen im Alter von 18–64 Jahren zu mindestens 150–300 min moderater oder 75–150 min intensiver körperlicher Aktivität pro Woche sowie zu muskelkräftigenden Aktivitäten an mindestens 2 Tagen. Sie sind heute maßgeblicher Referenzpunkt für die Bewertung des Aktivitätsniveaus von Bevölkerungen und Grundlage für nationale Gesundheitsberichterstattung und Präventionsplanung. Das Nichterreichen dieser Minimalkriterien wird mit „unzureichender körperlicher Aktivität“ beschrieben.

Anhand dieser Kriterien analysierte die aktuelle im *Lancet* publizierte Studie von Strain et al. [17] Daten aus 507 bevölkerungsbasierten Surveys in 197 Ländern, um globale Trends in der Entwicklung körperlicher Aktivität und Fortschritte bei der Erreichung des WHO-Ziels einer Reduktion des Anteils unzureichend aktiver Menschen um 15 % bis 2030 zu untersuchen. Ihre Ergebnisse dienen auch der WHO als Referenz zur Darstellung der weltweiten Prävalenz unzureichender körperlicher Aktivität. Für Deutschland weist die Studie für 2022 einen altersstandardisierten Anteil von lediglich 12 % unzureichend körperlich aktiver Erwachsener aus und zeichnet damit im internationalen Vergleich ein positives Bild [17]. Dieses Ergebnis fand auch in deutschen Medien breite Aufmerksamkeit, wie „Lage in Deutschland besser als weltweit“ (ZDFheute; [18]) oder „Menschen bewegen sich zu wenig, Deutsche raffen sich gut auf“ (*Rheinische Post*; [19]) zeigen.

Die Ergebnisse von Strain et al. stehen jedoch nur teilweise im Einklang mit nationalen Erhebungen und Studien zur körperlichen Aktivität. Während die NAKO-Gesundheitsstudie für den Zeitraum 2014–2019 ebenfalls einen Anteil von 12 % unzureichend körperlich aktiver Erwachsener berichtet, weisen die DKV-Reports für die Jahre 2018 bis 2025 Prävalenzen zwischen 15 % und 20 % aus [20, 21]. Die GEDA-Erhebungen des Robert Koch-Instituts berichten demgegenüber deutlich höhere Prävalenzen von 54,7 % (2014/2015) beziehungsweise 52 % (2019; [22, 23]).

Diese Diskrepanz zwischen positivem medialen Echo und den weniger positiven Ergebnissen weiterer Erhebungen birgt die Gefahr, dass politische Entscheidungsträger die Förderung von Bewegung in der Priorisierung zurückstufen oder Fördermittel kürzen – in der möglicherweise fälschlichen Annahme, das Problem sei weitgehend gelöst. Angesichts der Tatsache, dass in den vergangenen Jahren gezielt Programme wie die vom Bund finanzierte „ReStart“-Kampagne oder die „Nationalen Empfehlungen für Bewegung und Bewegungsförderung“ ins Leben gerufen wurden, wäre ein solcher Rückschritt besonders problematisch. Historische Beispiele wie die „Trimm-Dich“-Kampagne verdeutlichen, dass langfristig angelegte, breit verankerte und kontextsensibel gestaltete Präventionsstrategien in der Lage sind, große Teile der Bevölkerung zu erreichen und nachhaltige Veränderungen anzustoßen [15].

Vor diesem Hintergrund verfolgt der Beitrag das Ziel, die Erhebungsmethoden und Interpretationsansätze der *Lancet*-Studie kritisch zu beleuchten und in Relation zu anderen nationalen Datenquellen zu setzen. Zudem sollen methodische Unterschiede in der Datenerhebung und -auswertung sowie ihre Bedeutung für die Bewertung von Aktivitätsniveaus diskutiert werden. Ein besonderes Augenmerk liegt dabei auf generellen Herausforderungen und Limitationen der Messung und Interpretation (unzureichender) körperlicher Aktivität, um eine fundierte Grundlage für gesundheitspolitische Entscheidungen zu schaffen.

## Methodische Ansätze zur Erfassung und statistischen Aufbereitung körperlicher Aktivitätsdaten

### Datenerfassung

Um die unterschiedlichen Prävalenzraten unzureichender körperlicher Aktivität einordnen zu können, ist zunächst zu betrachten, auf welche Weise und mit welchen Instrumenten körperliche Aktivität in bevölkerungsbezogenen Studien erfasst wird. Unterschiede bestehen dabei insbesondere hinsichtlich der eingesetzten Messinstrumente, der Art der Datenerhebung, der Operationalisierung körperlicher Aktivität sowie der Auswertung und statistischen Verarbeitung der erhobenen Daten. Grundsätzlich kann zwischen selbstberichteten Verfahren und objektiven Messverfahren differenziert werden.

Selbstberichtete Verfahren basieren in der Regel auf standardisierten Fragebögen, während objektive Messverfahren überwiegend auf dem Einsatz von Akzelerometern (am Körper getragene Bewegungssensoren) beruhen. Zu den international am häufigsten eingesetzten Fragebogeninstrumenten zählen der von der WHO entwickelte Global Physical Activity Questionnaire (GPAQ; [24]), der International Physical Activity Questionnaire (IPAQ; [25]) sowie der European Health Interview Survey Physical Activity Questionnaire (EHIS-PAQ; [26]).

Die Fragebogeninstrumente unterscheiden sich hinsichtlich ihrer Konzeption und der Operationalisierung körperlicher Aktivität. Dies betrifft unter anderem die berücksichtigten Domänen körperlicher Aktivität, also die Lebensbereiche, in denen körperliche Aktivität stattfindet, sowie die Erfassung unterschiedlicher Intensitäten und die Auswertung der erhobenen Daten. Auch die Art der Datenerhebung kann variieren. Die Befragung erfolgt für gewöhnlich entweder in persönlichen Interviews (Face-to-Face) oder mittels schriftlicher beziehungsweise onlinebasierter Befragungen.

Neben Fragebogenverfahren kommen in einzelnen Studien auch Akzelerometer zum Einsatz. Auch zwischen den eingesetzten Geräten und den zur Datenverarbeitung verwendeten Algorithmen bestehen Unterschiede.

### Statistische Aufbereitung

Schließlich können die erhobenen Daten zusätzlich statistisch modelliert werden, um aus heterogenen Datensätzen konsistente Schätzungen der Prävalenz unzureichender körperlicher Aktivität abzuleiten. Ein solcher Ansatz wurde in der Studie von Strain et al. angewandt, indem hierarchische bayesianische Modelle eingesetzt wurden. Diese berücksichtigen Informationen aus unterschiedlichen Ebenen, etwa Ländern, Regionen, Altersgruppen oder Zeitpunkten, gleichzeitig und verknüpfen sie miteinander. Im Gegensatz zu klassischen Modellen behandeln sie fehlende oder schwache Daten nicht isoliert, sondern „leihen“ sich statistische Stärke aus vergleichbaren Kontexten (das sog. Borrowing Strength). Dadurch können stabilere und plausiblere Schätzungen entstehen, selbst wenn einzelne Länder oder Jahre nur wenige Datenpunkte liefern. Ein weiterer Vorteil dieser Vorgehensweise ist, dass Unsicherheiten explizit als Wahrscheinlichkeitsverteilungen quantifiziert werden, was eine differenzierte Einschätzung von Trends und Projektionen erlaubt. In der Studie wurde dieser Ansatz genutzt, um aus 507 teils heterogenen Umfragen, unterschiedlich in Zeit, Region und Erhebungsinstrument, konsistente Schätzungen für 197 Länder zu generieren.

Auch für Daten aus Deutschland wurde die Methode genutzt, um Trends über verschiedene Erhebungszeitpunkte hinweg zu modellieren und die Vergleichbarkeit unterschiedlicher Datensätze herzustellen. Analysiert wurden Eurobarometer- und DKV-Erhebungen von 2002 bis 2022. Während die Eurobarometer-Daten zunächst auf dem IPAQ, später auf einem eigens konstruierten, an den IPAQ angelehnten Fragebogen basierten, nutzten die DKV-Erhebungen durchgehend den GPAQ. Alle Datensätze wurden nach dem WHO-Standard oder angepassten, harmonisierten Verfahren ausgewertet und an das GPAQ-Referenzprotokoll angeglichen. Bei der Berechnung der Prävalenz unzureichender körperlicher Aktivität wurden geschlechts- und altersspezifische Gewichtungen, komplexe Stichprobendesigns sowie Korrekturmodelle berücksichtigt [17].

## Erhebungen zur körperlichen Aktivität in Deutschland im Vergleich

**Abb. 1:**
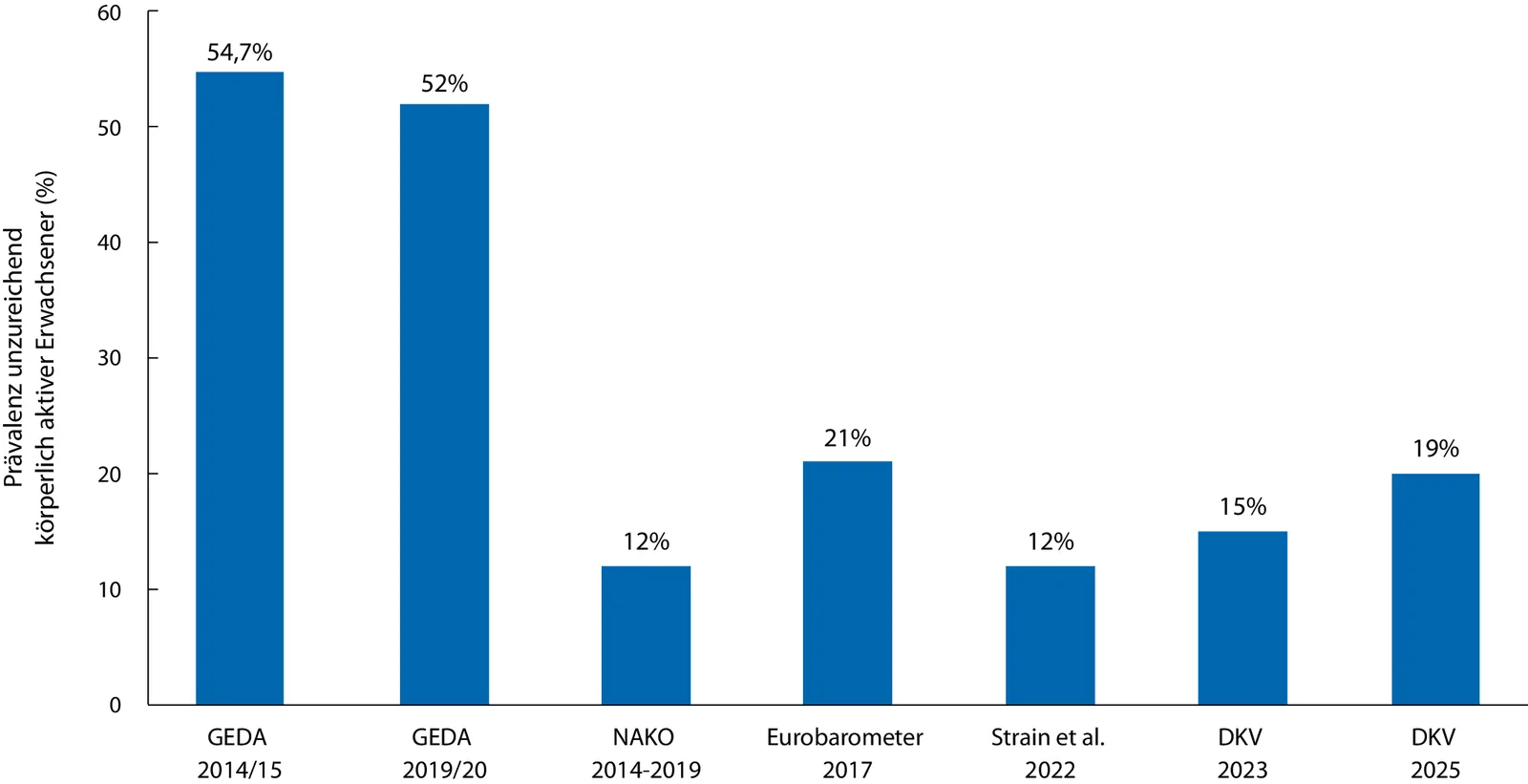
Eigene Darstellung zu Prävalenz unzureichender körperlicher Aktivität (in %) und Erhebungszeitraum. (Nach GEDA 2014/2015 [27], GEDA 2019/2020 [22], NAKO [28], Eurobarometer 2017 gemäß Nikitara et al. [29], Strain et al. [17], DKV 2023 [20] und DKV 2025 [21])

Die zuvor genannten Erhebungen (NAKO-Gesundheitsstudie, DKV-Reports 2018–2025, GEDA 2014/2015 und GEDA 2019/2020) unterscheiden sich hinsichtlich ihrer Methodik, insbesondere bezüglich der eingesetzten Erhebungsinstrumente, der Art der Datenerhebung sowie der Auswertung der erhobenen Daten. Im Folgenden werden die wichtigsten nationalen und internationalen Erhebungen hinsichtlich dieser Merkmale sowie der jeweils berichteten Prävalenz unzureichender körperlicher Aktivität vorgestellt (Abb. 1). Die hier berichteten Prävalenzen unzureichender körperlicher Aktivität beziehen sich auf die Kriterien zur Ausdaueraktivität, da die von der WHO zusätzlich angeführte Vorgabe von muskelkräftigender Aktivität an mindestens 2 Tagen pro Woche nur in einzelnen Erhebungen zusätzlich erfasst und berichtet wird.

Die Studie „Gesundheit in Deutschland aktuell“ (*GEDA*) ist eine wiederkehrende Gesundheitsbefragung des Robert Koch-Instituts (RKI) mit dem Ziel, repräsentative Daten zum Gesundheitsverhalten der erwachsenen Bevölkerung in Deutschland zu erheben. Zur Erfassung der körperlichen Aktivität wird der im Rahmen des „European Health Interview Survey“ entwickelte EHIS-PAQ eingesetzt. Die Datenerhebung erfolgt im Rahmen bevölkerungsrepräsentativer Gesundheitsbefragungen. Während für die Erhebung 2014/2015 Papier- und Onlinefragebögen genutzt wurden, wurden 2019/2020 computerbasierte Telefoninterviews durchgeführt. Für das Jahr 2019 berichtet das RKI eine Prävalenz unzureichender körperlicher Aktivität gemäß den WHO-Empfehlungen zur Ausdaueraktivität von 52 %. In der Erhebung GEDA 2014/2015 lag dieser Wert bei 54,7 % [22, 27].

Der *DKV-Report *„Wie gesund lebt Deutschland?“ ist eine regelmäßig durchgeführte bevölkerungsbezogene Erhebung zum Gesundheitsverhalten der erwachsenen Bevölkerung in Deutschland. Die körperliche Aktivität wird mittels Onlinebefragungen unter Verwendung des von der WHO entwickelten GPAQ erfasst. Für die Erhebungsjahre 2018, 2021, 2023 und 2025 wurden Prävalenzen unzureichender körperlicher Aktivität von 20 %, 19 %, 15 % beziehungsweise 19 % berichtet [20, 21].

Die *NAKO-Gesundheitsstudie* ist eine bundesweite bevölkerungsbasierte Langzeitstudie. An der von 2014 bis 2019 durchführten Basisuntersuchung nahmen rund 205.000 Personen teil. Neben zahlreichen weiteren gesundheitsbezogenen Parametern wurde auch die körperliche Aktivität erfasst. Neben der Nutzung des GPAQ als selbstberichtetes Verfahren kamen auch Akzelerometer zum Einsatz. Der GPAQ wurde über einen elektronischen Touchscreen-Fragebogen von den Teilnehmenden eigenständig ausgefüllt. Dieses Vorgehen reduziert den Umfang persönlicher Interviews und unterscheidet sich sowohl von klassischen Face-to-Face-Interviews als auch von webbasierten Onlinebefragungen. Bisher liegen noch keine Publikationen vor, welche akzelerometerbasierte Daten im Hinblick auf unzureichende körperliche Aktivität systematisch aufarbeiten und nationale Prävalenzen berichten. Erste Analysen anhand mittels GPAQ ermittelter Daten berichten für den Erhebungszeitraum 2014 bis 2019 eine Prävalenz unzureichender körperlicher Aktivität von rund 12 % [28].

Der *Eurobarometer* ist eine regelmäßig durchgeführte Bevölkerungsbefragung der Europäischen Kommission. Die Datenerhebung erfolgt im Rahmen persönlicher Face-to-Face-Interviews. Während frühere Erhebungswellen IPAQ verwendeten, kommt in neueren Erhebungen ein eigens für den Eurobarometer entwickelter Fragebogen zum Einsatz. Da dieser nicht unmittelbar auf die WHO-Empfehlungen zur körperlichen Aktivität ausgerichtet ist, müssen die erhobenen Daten vor einer vergleichbaren Auswertung zunächst transformiert werden. Für Deutschland wurde auf Grundlage der Erhebung aus dem Jahr 2017 nach entsprechender Transformation eine Prävalenz unzureichender körperlicher Aktivität von 21 % berichtet [29].

Die im *Lancet* publizierte *Studie von Strain et al.* [17] unterscheidet sich von den zuvor beschriebenen Erhebungen dadurch, dass sie keine eigenständige Datenerhebung durchführte, sondern bereits vorliegende bevölkerungsrepräsentative Erhebungen zusammenführte. Für Deutschland wurden, wie erwähnt, hierfür Daten des Eurobarometers sowie der DKV-Reports aus den Jahren 2002 bis 2022 herangezogen und mittels hierarchisch bayesianischer Modelle berechnet. Somit stellen die dort berichteten Prävalenzen modellierte Schätzwerte dar und entsprechen nicht den ursprünglichen Ergebnissen der einzelnen Erhebungen. Der Anteil der unzureichend körperlich Aktiven in Deutschland sank im Modell von Strain et al. von 20,3 % im Jahr 2000 auf 16,6 % im Jahr 2010 und weiter auf 12 % im Jahr 2022. Für 2030 wird ein Wert von 9,5 % prognostiziert; Deutschland ist demnach mit hoher Sicherheit auf gutem Weg, die WHO-Vorgaben zu erreichen. Im europäischen Vergleich (der durch Strain et al. ermittelten Prävalenzen) reiht sich Deutschland damit im Spitzenfeld um Länder wie Schweden, die Niederlande oder Dänemark ein und hebt sich deutlich von den eher inaktiven Ländern im europäischen Umfeld wie etwa Italien (40 % unzureichend körperlich Aktive) ab. Auch im Vergleich zur durchschnittlichen Prävalenz unzureichend körperlich Aktiver in der Gruppe der westlichen Hocheinkommensländer (27 %) schneidet Deutschland gut ab [17].

## Methodische Limitationen bei der Erfassung körperlicher Aktivität

Die teilweise erheblichen Unterschiede zwischen den vorgestellten Erhebungen lassen sich möglicherweise durch die nachfolgend dargestellten methodischen Unterschiede erklären. Diese sind daher bei der Interpretation und Vergleichbarkeit der berichteten Prävalenzschätzungen zu berücksichtigen (Tab. 1).

**Tab. 1:** Übersicht zentraler methodischer Merkmale der diskutierten Studien

Studie	Erhebungszeitraum	Instrument	Erhebungsmodus	Aktivitätsdomänen	Statistische Aufbereitung
GEDA 2014/15	2014–2015	EHIS-PAQ	Papier‑/Online-Fragebogen	Freizeit, Transport (keine Arbeitsaktivität)	Standardauswertung
GEDA 2019/20	2019–2020	EHIS-PAQ	Telefoninterview	Freizeit, Transport (keine Arbeitsaktivität)	Standardauswertung
DKV-Reports	2018–2025	GPAQ	Onlinebefragung	Arbeit, Transport, Freizeit	Standardauswertung
NAKO	2014–2019	GPAQ	Elektronischer Fragebogen	Arbeit, Transport, Freizeit	Standardauswertung
Eurobarometer	2017	Modifizierter IPAQ	Face-to-Face	Arbeit, Transport, Freizeit	Transformation auf WHO-Kriterien
Strain et al.	2000–2022	GPAQ/IPAQ und weitere	Keine eigene Datenerhebung	Abhängig von Ausgangsstudien	Hierarchisch bayesianische Modelle

Bereits die Wahl des Erhebungsinstruments kann die berichteten Prävalenzen beeinflussen. Während die DKV-Reports sowie die NAKO-Gesundheitsstudie den GPAQ verwenden, basiert der Eurobarometer auf einem modifizierten, an den IPAQ angelehnten Fragebogen. Die GEDA-Studien nutzen hingegen EHIS-PAQ. Obwohl alle 3 Instrumente der Erfassung körperlicher Aktivität dienen, unterscheiden sie sich hinsichtlich ihrer Konzeption und der Operationalisierung der WHO-Empfehlungen zur körperlichen Aktivität:

Der IPAQ und der GPAQ verfolgen einen vergleichbaren Ansatz. Beide erfassen körperliche Aktivität in verschiedenen Domänen (insbesondere Arbeit, Fortbewegung und Freizeit) und berechnen daraus den wöchentlichen Energieaufwand in Form von Metabolic-Equivalent-of-Task-(MET-)Minuten. Moderate und intensive körperliche Aktivität werden entsprechend ihrer Intensität mit standardisierten MET-Werten (4 bzw. 8 MET) gewichtet. Die MET-Minuten ergeben sich aus der Multiplikation des jeweiligen MET-Wertes mit Dauer und Häufigkeit der Aktivität und werden anschließend über die einzelnen Domänen summiert. Dadurch können moderate und intensive körperliche Aktivität in einer gemeinsamen Maßeinheit ausgedrückt werden. Ein Gesamtwert von mindestens 600 MET-Minuten pro Woche entspricht dabei einer äquivalenten Kombination der WHO-Empfehlung von mindestens 150 min moderater oder 75 min intensiver körperlicher Aktivität pro Woche beziehungsweise einer entsprechenden Kombination beider Intensitäten. Unterschiede liegen etwa darin, dass beim IPAQ die körperliche Aktivität der letzten 7 Tage erfragt wird, während beim GPAQ die Aktivität einer gewöhnlichen Woche geschätzt wird. Außerdem gibt es Unterschiede in der Kategorisierung der Intensität von Aktivitäten, beispielsweise dem Gehen [24, 25, 30].

Der EHIS-PAQ verfolgt demgegenüber einen anderen Ansatz. Zwar werden auch hier körperliche Aktivität in den Domänen Arbeit, Transport und Freizeit sowie muskelkräftigende Aktivitäten erfasst, jedoch dient der Fragebogen nicht der Berechnung eines domänenübergreifenden MET-Gesamtscores, sondern der Ableitung bevölkerungsbezogener WHO-Indikatoren für das europäische Gesundheitsmonitoring. Für die Operationalisierung der WHO-Empfehlungen werden dabei Angaben zur freizeitbezogenen körperlichen Aktivität sowie zum Radfahren als aktive Fortbewegung berücksichtigt, während arbeitsbezogene körperliche Aktivität nicht in die Berechnung des Indikators eingeht [26, 27]. Dieser Ansatz trägt der zunehmenden Evidenz Rechnung, dass körperliche Aktivität je nach Domäne unterschiedliche gesundheitliche Wirkungen entfalten kann. Während freizeitbezogene körperliche Aktivität konsistent mit positiven gesundheitlichen Effekten assoziiert ist, zeigen sich diese Zusammenhänge für berufliche körperliche Aktivität nicht in gleicher Weise (Physical Activity Paradox; [31]).

Gemeinsam ist den Erhebungen, dass sie überwiegend auf Selbstangaben der Teilnehmenden beruhen. Standardisierte Fragebogeninstrumente sind leicht anzuwenden und ermöglichen bevölkerungsrepräsentative Erhebungen sowie internationale Vergleiche. Im Vergleich zu objektiven Messverfahren wie Akzelerometern überschätzen sie die tatsächliche körperliche Aktivität jedoch häufig und weisen eine geringere Validität auf [32–35]. Typische Probleme ergeben sich insbesondere durch Erinnerungsverzerrungen und sozial erwünschte Antworten, durch Schwierigkeiten der Befragten bei der Abgrenzung von moderater gegenüber intensiver Aktivität sowie durch kultur- und sprachbedingte Unterschiede in der Interpretation einzelner Fragen [36–38].

Zusätzlich erschweren Unterschiede in der Durchführung der Erhebungen die Vergleichbarkeit der Ergebnisse. Face-to-Face-Befragungen, wie sie im Eurobarometer eingesetzt werden, liefern zwar häufig repräsentativere Stichproben, sind jedoch stärker durch sozial erwünschtes Antwortverhalten geprägt und können dadurch zu einer Überschätzung der körperlichen Aktivität führen. Onlinebefragungen, wie sie den DKV-Reports zugrunde liegen, ermöglichen demgegenüber eine anonyme Teilnahme und können dadurch ehrlichere Angaben begünstigen. Gleichzeitig weisen sie häufiger Selektionsverzerrungen auf, da internetaffine und jüngere Personen überrepräsentiert sein können [39, 40].

Die bislang beschriebenen Einflussfaktoren betreffen hauptsächlich selbstberichtete Verfahren. Objektive Bewegungsdaten mittels Akzelerometern unterliegen demgegenüber nicht den genannten Limitationen wie Erinnerungsverzerrungen, sozial erwünschtem Antwortverhalten und subjektiven Einschätzungsfehlern. Gerade in groß angelegten bevölkerungsrepräsentativen Studien ist ihr Einsatz jedoch mit erheblich höheren Kosten und organisatorischem Aufwand verbunden. Darüber hinaus unterliegen auch objektive Messverfahren Limitationen. So können unterschiedliche Geräte oder bei gleichem Gerät verschiedene Algorithmen zur Datenverarbeitung zu voneinander abweichenden Ergebnissen führen [41]. Auch methodische Entscheidungen beeinflussen die Prävalenzschätzungen. Dazu zählt etwa die Nichtberücksichtigung von Aktivitätsepisoden unterhalb 10 min Dauer, die auch in den genannten Fragebogeninstrumenten verankert ist, in den aktuellen WHO-Kriterien jedoch nicht mehr vorgesehen ist. Zudem erfassen Akzelerometer körperliche Aktivität nicht domänenspezifisch und erlauben daher nur eingeschränkte Aussagen über Art und Gesundheitsförderlichkeit der jeweiligen Aktivität. Dies ist insbesondere vor dem Hintergrund des bereits erwähnten Physical Activity Paradox relevant.

Die beschriebenen Unterschiede zwischen verschiedenen Erhebungen erschweren eine unmittelbare Vergleichbarkeit der berichteten Prävalenzen. Um diese Heterogenität zumindest teilweise auszugleichen, können statistische Modellierungsverfahren zum Einsatz kommen. Die Studie von Strain et al. nutzte hierzu hierarchische bayesianische Modelle, um Unterschiede zwischen Erhebungsinstrumenten, Erhebungszeiträumen und Datenverfügbarkeit statistisch zu berücksichtigen und international vergleichbare Prävalenzschätzungen zu generieren. Diese Vorgehensweise ermöglicht internationale Vergleiche sowie die Analyse langfristiger Trends, setzt jedoch umfangreiche Harmonisierungsschritte voraus, die mit Unsicherheiten verbunden sind. Wie Strain et al. hervorheben, können Unterschiede zwischen den verwendeten Erhebungsinstrumenten sowie die überwiegend auf Selbstangaben beruhenden Daten trotz statistischer Anpassungen zu systematischen Verzerrungen führen. Dadurch besteht das Risiko, dass die Prävalenz unzureichender körperlicher Aktivität in einzelnen Ländern sowohl über- als auch unterschätzt wird [17]. Die Ergebnisse solcher modellbasierten Analysen sollten daher stets vor dem Hintergrund der zugrunde liegenden Daten interpretiert werden.

## Körperliche Aktivität vergleichbar erfassen und berichten

Angesichts der vielfältigen methodischen Herausforderungen stellt sich die Frage, wie körperliche Aktivität künftig möglichst valide und vergleichbar erfasst und berichtet werden kann. Hier besteht weiterhin Diskussions- und Harmonisierungsbedarf. Dies betrifft beispielsweise die Berücksichtigung der von der WHO empfohlenen muskelkräftigenden Aktivitäten an mindestens 2 Tagen pro Woche und die zumeist verwendete 10-Minuten-Mindestdauer einzelner Aktivitätsepisoden, obwohl diese in den aktuellen WHO-Empfehlungen nicht mehr enthalten ist, sowie den Ein- oder Ausschluss arbeitsbezogener körperlicher Aktivität. Insbesondere Letzteres dürfte wesentlich zu den unterschiedlichen Prävalenzschätzungen der hier betrachteten Erhebungen beigetragen haben.

Auch der vermehrte Einsatz von kombinierten Erhebungsmethoden, also Akzelerometern und selbstberichteten Fragebögen gemeinsam, scheint empfehlenswert. So können instrumentenspezifische Verzerrungen klarer eingeordnet werden und möglicherweise langfristig multimodale Erhebungs- und Auswertungsmethoden entwickelt werden, welche die Vorteile beider Methoden verbinden. Beispielsweise könnten parallel erhobene Akzelerometerdaten dazu genutzt werden, die Zuordnung selbstberichteter Aktivitäten zu moderaten und intensiven Belastungen besser zu validieren und so bestehende Fragebogeninstrumente evidenzbasiert weiterzuentwickeln. Umgekehrt können selbstberichtete Fragebögen die Einordnung akzelerometerbasierter Aktivitätsdaten in unterschiedliche Domänen wie Freizeit, Arbeit oder Transport ermöglichen und so zu einer differenzierteren Interpretation beitragen.

## Fazit

Die Analyse nationaler und internationaler Erhebungen zur körperlichen Aktivität verdeutlicht, dass zentrale Unterschiede in Methodik (z. B. objektive versus subjektive Erfassung), Messinstrument (z. B. EHIS-PAQ, GPAQ) und Auswertung (MET-Scores, Modellierungen) zu teilweise erheblich divergierenden Ergebnissen führen können. Modellierte globale Schätzungen, wie sie Strain et al. (2024) vorlegen, zeichnen für Deutschland ein deutlich günstigeres Aktivitätsbild als nationale Gesundheitsbefragungen wie GEDA. Diese Differenzen sind keine Widersprüche im engeren Sinne, sondern spiegeln zugrunde liegende Unterschiede der Datenerhebung und Bewertung wider.

Für die gesundheitspolitische Einordnung sind diese methodischen Aspekte jedoch von entscheidender Bedeutung. Denn ein auf statistischen Näherungen basierendes positives Aktivitätsbild kann zu Fehleinschätzungen führen, wenn daraus eine vermeintlich geringe Dringlichkeit für Bewegungsförderung abgeleitet wird. Nationale Datenquellen, die eine höhere Prävalenz unzureichender körperlicher Aktivität verzeichnen, verweisen hingegen auf einen weiterhin substanziellen Förderungsbedarf.

Die Sicherstellung konsistenter, belastbarer Bewegungsdaten bleibt daher eine Kernaufgabe zukünftiger Forschung und Gesundheitsberichterstattung. Neben einer verstärkten Förderung der Entwicklung und Implementierung multimodaler Datenerhebungsstrategien, die Selbstbericht und objektive Messverfahren kombinieren, besteht weiterhin Forschungsbedarf hinsichtlich einer einheitlichen Operationalisierung der WHO-Mindestkriterien. Hierzu gehört insbesondere die kritische Auseinandersetzung mit methodischen Fragen, etwa der Berücksichtigung arbeitsbezogener körperlicher Aktivität.

Nur durch valide, vergleichbare und fortlaufend erhobene Aktivitätsdaten lässt sich einschätzen, ob Deutschland tatsächlich zu den „aktiveren“ Nationen zählt – oder ob die Herausforderung des Bewegungsmangels lediglich durch methodische Unterschiede unterschätzt wird. Solange keine neuen, methodisch einheitlichen und belastbaren Erhebungen vorliegen, gilt es aus unserer Sicht, die bestehenden Programme zur Bewegungsförderung nachhaltig fortzuführen.
